# Extraction and Chromatographic Determination of Shikimic Acid in Chinese Conifer Needles with 1-Benzyl-3-methylimidazolium Bromide Ionic Liquid Aqueous Solutions

**DOI:** 10.1155/2014/256473

**Published:** 2014-03-23

**Authors:** Fengli Chen, Kexin Hou, Shuangyang Li, Yuangang Zu, Lei Yang

**Affiliations:** Key Laboratory of Forest Plant Ecology, Ministry of Education, Northeast Forestry University, Hexing Road 26, Harbin 150040, China

## Abstract

An ionic liquids-based ultrasound-assisted extraction (ILUAE) method was successfully developed for extracting shikimic acid from conifer needles. Eleven 1-alkyl-3-methylimidazolium ionic liquids with different cations and anions were investigated and 1-benzyl-3-methylimidazolium bromide solution was selected as the solvent. The conditions for ILUAE, including the ionic liquid concentration, ultrasound power, ultrasound time, and liquid-solid ratio, were optimized. The proposed method had good recovery (99.37%–100.11%) and reproducibility (RSD, *n* = 6; 3.6%). ILUAE was an efficient, rapid, and simple sample preparation technique that showed high reproducibility. Based on the results, a number of plant species, namely, *Picea koraiensis, Picea meyeri, Pinus elliottii,* and *Pinus banksiana,* were identified as among the best resources of shikimic acid.

## 1. Introduction

**Figure 1:**
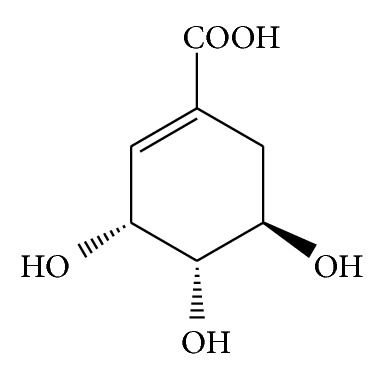
The molecular structure of shikimic acid.

Shikimic acid is a white crystalline cyclitol, which is a precursor material for industrial synthesis of the important drug, oseltamivir phosphate (Tamiflu), which is an effective drug against the H5N1 influenza virus [1], especially if administered early. According to the in vitro and in vivo studies, shikimic acid and its derivatives possess many pharmacological effects, such as antithrombotic [2], anticoagulant [3], anti-inflammatory [4], analgesic [5], antioxidant [6], anticancer [7], and antibacterial effects [8]. Serious health threats have promoted social development and the concerns over an outbreak of the disease have increased the need for shikimic acid [9]. Shikimic acid (Figure 1) was first isolated from the fruit of* Illicium religiosum* by Eykman in 1885 [10]. Currently, most of the shikimic acid required by the pharmaceutical industry was obtained from the fruits of Chinese plant star anise (*I. verum*) [11]. The fruits of this tree were reported that the content of shikimic acid ranged from 2 to 7% [12]; however, only* I. verum* resource could not satisfy its growing market demand as growing difficulties. Potential alternative resources of shikimic acid are thus urgently required. Shikimic acid is a widely occurring primary plant metabolite, which has been identified in some conifer needles with relative abundance distribution.

For extract preparation, selection of an appropriate extraction method is a key consideration. The extraction of shikimic acid has been accomplished by several extraction methods in the past, such as maceration extraction [13], homogenate extraction [14], and heat reflux extraction [15] with acidic water [16, 17], methanol [15], ethanol [18, 19],* n*-butanol [20], isopropanol [21], and some mixtures as solvents [22]. However, the main disadvantage of traditional extraction methods lies in the complicated working procedure which increased the energy consumption and repeated distillation processes can prolong the heating time and lead to unsatisfactory recoveries. Moreover, these organic solvents used are problematic in the extraction of shikimic acid because of their toxicity volatility and flammability.

Nowadays, people pay increasing attention on development and use of environmentally friendly methods. Ultrasound assisted extraction (UAE) is an extraction technique that can offer high reproducibility in simplified manipulation, shorten extraction time, reduce solvent consumption, and lower energy input, which has been widely used to extract analytes from many matrixes [23–27]. Ultrasound enhancement of extraction is attributed to the disruption of cell walls, particle-size reduction, and the enhancement on the mass transfer of the cell content to the solvent caused by the collapse of the bubbles produced by cavitations [28, 29]. It is obvious that finding an effective method for extraction of shikimic acid is very important.

Ionic liquids are composed of organic cations and inorganic or organic anions and are liquid near room temperature. In recent years, ionic liquids have been used as attractive “green” alternatives to conventional volatile organic solvents in various applications, particularly in separation science [30, 31]. Due to their unique chemical and physical properties, such as negligible vapor pressure, wide liquid range, good stability, tunable viscosity, good miscibility in water and organic solvents, and good solubility and extractability for various organic compounds and remarkable advantage of easy to be controlled over conventional solvents [32], they have been found with a big field in microextraction techniques, both in liquid phase [33] and in solid phase [31, 34]. Meanwhile, ILs have been used as extraction solvent to dispose of plant materials for extraction of components, such as alkaloid [35, 36], procyanidins [37, 38], flavonoid [26], glycoside [27], lignan [39], and coumarin [25]. Recently ionic liquid 1-butyl-3-methylimidazolium chloride has been used for extracting shikimic acid from* Ginkgo biloba* leaves due to dissolve cellulose at high temperature 150°C [40]. However, to our knowledge, there are no reports on the extraction of cyclitol using ionic liquid aqueous solution utilizing room temperature technology.

The aim of the present paper is to (i) develop a rapid and effective ionic liquid-based ultrasound-assisted extraction (ILUAE) approach for the extraction of shikimic acid from conifer needles. Herein we described our investigations on the performances of various ionic liquids with different cations and anions in an ILUAE method. It was found that parameters including the ionic liquid concentration, solid-liquid ratio, ultrasound power, and time were influential on the final yield, and these parameters were optimized systematically. Here high-performance liquid chromatography was used to quantify the content of shikimic acid. The ILUAE approach developed here was compared with conventional extraction approaches. Moreover, the proposed method was validated in stability, repeatability, and recovery experiments; (ii) a large number of conifer needles of Chinese origin with respect to their shikimic acid contents were selected to find new potential resources.

## 2. Experimental

### 2.1. Chemicals

Reference compound of shikimic acid was purchased from Sigma-Aldrich Inc. (St. Louis, MO, USA). Methanol and sulfuric acid of HPLC grade were purchased from J & K Chemical Ltd. (Beijing, China). All ionic liquids ([C_4_mim]Cl, [C_4_mim]BF_4_, [C_4_mim]NO_3_, [C_2_mim]Br, [C_3_mim]Br, [C_4_mim]Br, [C_5_mim]Br, [C_6_mim]Br, [C_8_mim]Br, [C_10_mim]Br, and [Bzmim]Br, where C_2_ = 1-ethyl, C_3_ = 1-propyl, C_4_ = 1-butyl, C_5_ = 1-pentyl, C_6_ = 1-hexyl, C_8_ = 1-octyl, C_10_ = 1-decyl, Bz = 1-benzyl, and mim = 3-methylimidazolium) were bought from Shanghai Cheng Jie Chemical Co., Ltd. (Shanghai, China). All other reagents were of analytical grade and purchased from Tianjin Chemical Reagents Co. (Tianjin, China). Deionized water was purified by a Milli-Q water purification system (Millipore, MA, USA). All solutions and samples prepared for HPLC were filtered through 0.45 *μ*m nylon membranes (Millipore, MA, USA) before use.

### 2.2. Sample Preparation

Samples of conifer needles were collected on September from eight different regions in China. The main climate data were shown in Table S1 (see Supplementary Material available online at http://dx.doi.org/10.1155/2014/256473). The collected materials were authenticated by Professor Shao-quan Nie of the Key Laboratory of Forest Plant Ecology, Ministry of Education, Northeast Forestry University, China. Voucher specimens of collected plant species were deposited in the herbarium of this facility. The conifer needles were removed of mechanical impurities by manual sorting and air-dried at room temperature and then were ground to a fine powder using a laboratory mill and passed through a 60 mesh sieve, to provide homogeneous powders for the analysis. Powdered materials were stored in closed desiccators before using and the same batch of samples were used here in the experiments.

### 2.3. Apparatus

The extraction procedure was carried out in an ultrasound-assisted extraction unit (KQ-250DB, Kunshan Ultrasound Equipment Co., Ltd., Jiangsu, China). The bath is a rectangular container (23.5 × 13.3 × 10.2 cm), where 50 kHz transducers are annealed at the bottom. The bath power rating is 250 W on the scale of 0–100%. The high-performance liquid chromatography system (Waters, USA) was equipped with a 1525 binary HPLC pump, a 717 plus autosampler, a 717 automatic column temperature control box, and a 2487 dual absorbance detector. Chromatographic separation was performed on an Aichrom Bond-AQ C18 reversed-phase column (4.6 mm × 250 mm, 5 µm, Eka Nobel, Sweden).

### 2.4. HPLC Analysis and Quantification

Shikimic acid was dissolved in methanol to yield the stock solutions at a concentration of 2 mg/mL. The standard stock solution was stored at 4°C and diluted with methanol to the required concentration before direct analysis by HPLC.

The 0.01% sulfuric acid aqueous solution was used as mobile phase and the isocratic elution was used for separation. For HPLC analysis, the flow rate was 1.0 mL/min, the injection volume was 10 *μ*L, the UV detection wavelength was 213 nm for shikimic acid, the column temperature was 30°C, and the running time was 20 min. Under these chromatographic conditions, the retention time of shikimic acid was 5.9 min and the shikimic acid was resolved sufficiently to give baseline separation. Shikimic acid of 85 kinds of conifer needles was identified by comparing the retention time with standard solution.

### 2.5. Ionic Liquid-Based Ultrasound-Assisted Extraction (ILUAE)

1 g of dried* Picea meyeri *sample powders was soaked in various volume ionic liquid aqueous solutions in a 100 mL flask which was then partially suspended in the ultrasound bath. The suspension was then extracted by UAE, with temperature control achieved by the replacement of inlet and outlet water to avoid water temperature rises. 11 kinds of ionic liquids [C_4_mim]Cl, [C_4_mim]BF_4_, [C_4_mim]NO_3_, [C_2_mim]Br, [C_3_mim]Br, [C_4_mim]Br, [C_5_mim]Br, [C_6_mim]Br, [C_8_mim]Br, [C_10_mim]Br, and [Bzmim]Br (composed of different cations and anions) were studied to select the optimum ionic liquid for extraction of shikimic acid from* P. meyeri* needles. The optimum anion, cation, concentration of ionic liquid, ultrasound power, time for ultrasound treatment, and liquid-solid ratio were systematically studied through comparison of the extraction yield of shikimic acid. After each extraction, 1 mL of extract obtained was cooled to room temperature rapidly and filtered through a 0.45 µm membrane (Guangfu Chemical Reagents Co.,Tianjin, China) for subsequent HPLC analysis.

The extraction yield of shikimic acid was determined as follows:

(1) Yield(mg/g)=mean  mass  of  shikimic  acid  in  conifer  needles(mg)mean  mass  of  the  conifer  needles(g).

The mean mass of shikimic acid in conifer needles was determined by HPLC analysis of three samples, respectively. The mean mass of the conifer needles was the average mass of three samples before being extracted.

### 2.6. Optimization of ILUAE by RSM

The Design Expert (Version 7.0, Stat-Ease Inc., Minneapolis, MN, USA) software was used for experimental design, data analysis, and model building. A Box-Behnken design was used to determine the response pattern and then to establish a model. The ultrasound time, ultrasound power, and liquid-solid ratio were chosen as the key variables based on the results of preliminary experiments and were designated *X*_1_, *X*_2_, and *X*_3_, respectively, as shown in Table 1. Five replicates at the center of the design were used for the estimation of a pure-error sum of squares. Experiments were randomized to maximize the effects of unexplained variability, due to extraneous factors, in the observed responses. A quadratic equation was used for this model as follows:

(2) $$\begin{array}{c} Y=\beta_{0}+\sum_{i=1}^{3}\beta_{i}X_{i}+\sum_{i=1}^{3}\beta_{ii}X_{i}^{2}+\sum_{i=1}^{2}\sum_{j=i+1}^{3}\beta_{ij}X_{i}X_{j}, \end{array}$$

where *Y* is the estimated response; *β*_0_, *β*_*i*_, *β*_*ii*_, and *β*_*ij*_ are the regression coefficients for intercept, linearity, square, and interaction, respectively; and *X*_1_, *X*_2_, and *X*_3_ are the independent variables.

### 2.7. Method Validation

After optimization of the extraction, the method was validated in terms of linearity, limit of detection (LOD) and quantification (LOQ), reproducibility, stability, and recovery under the above optimized conditions.

Calibration curves were obtained using a series of standard solutions containing the shikimic acid at six different concentration levels (0.01, 0.2, 0.4, 0.6, 0.8, and 1.0 mg/mL) under the same HPLC conditions for conifer needles extraction. Each concentration was injected six times to ensure that accurate and reproducible responses have been generated. LOD for the determination of shikimic acid under the present chromatographic conditions was determined on the standard deviation of *y*-intercepts of the regression lines (*α*) and the slope (*S*), using the following equation: LOD = 3.3*α*/*S*. LOQ was calculated by the equation LOQ = 10*α*/*S*.

The stability of shikimic acid was determined using standards dissolved in 0.5 mol/L [Bzmim]Br and extracted by UAE under the optimum conditions (ultrasound power = 170 W, ultrasound time = 39 min, and liquid-solid ratio = 8.3 : 1 mL/g). In order to evaluate the recovery of shikimic acid, a standard solution of shikimic acid was added to* Picea meyeri* needle samples at three different concentrations and extractions were performed by UAE using the optimum conditions ([Bzmim]Br = 0.5 mol/L, ultrasound power = 170 W, ultrasound time = 39 min, and liquid-solid ratio = 8.3 : 1 mL/g). The initial concentration of shikimic acid determined by HPLC was 2.10 mg/mL. The recovery of shikimic acid was taken as an indicator of the stability of shikimic acid under the extraction conditions. Five samples of the same weight (1.0 g) were processed under optimum extraction conditions to determine the reproducibility of the extraction method.

### 2.8. Reference and Conventional Extraction Methods

Pure water, 0.5 mol/L NaBr, and 80% ethanol were selected as reference solvents for extraction of shikimic acid from* Picea meyeri* needles by UAE. The extraction experiments were operated under the optimum conditions except for the solvent type. The dried* Picea meyeri* needle samples (1.0 g) were mixed with 8.3 mL of the above solvents and the suspension was then extracted for 39 min by UAE at 170 W ultrasound power. For heat reflux extraction (HRE), 80% ethanol and pure water were selected as solvents and the main technical parameters used were listed in Table 3. The extract was filtered through a nylon membrane prior to HPLC analysis.

## 3. Results and Discussion

### 3.1. Screening of the Ionic Liquid-Based Extracting Solvent

**Figure 2:**
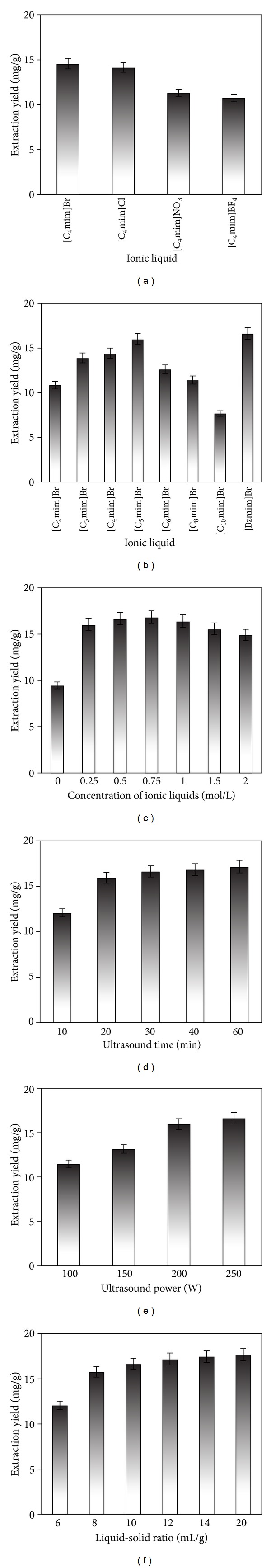
Effects of the ionic liquids anions (a), cations (b), concentration of [Bzmim]Br (c), ultrasound time (d), ultrasound power (e), and liquid-solid ratio (f) on the extraction yield of shikimic acid.

As the structures of ionic liquids determine their physical and chemical properties, ionic liquids have great impact on extracting analytes [41]. To find out the optimal ionic liquid and evaluate its performance on the extraction of shikimic acid, the effects of changing the anion and the alkyl chain length of the cation of 1-alkyl-3-methylimidazolium-type ionic liquids on the extraction yield were studied and the general trends observed were described below. The experimental results were shown in Figure 2.

#### 3.1.1. Anion Effect

It is well known that the choice of anion affects water miscibility of ionic liquids [42]. Thus, in order to investigate the effects of ionic liquids with different anions on extraction yield, 1-butyl-3-methylimidazolium ionic liquids with four kinds of anions (Cl^−^, Br^−^, NO_3_^−^, and BF_4_^−^) were studied by ILUAE. The extraction results of four different ionic liquid aqueous solutions with the same cation but different anions were shown in Figure 2(a), from which we can conclude that the anion of ionic liquids influenced the extraction yield of target analyte and [C_4_mim]Br and [C_4_mim]Cl were more efficient than other two ionic liquids, which was likely related to the great effect of anion on the overall hydrogen bond basicity of the 1-butyl-3-methylimdazolium based ionic liquids [41]. Br^−^ series was chosen as extraction solvent in the following studies because the yield differences of shikimic acid were not significant among [C_4_mim]Br and [C_4_mim]Cl solution.

#### 3.1.2. Cations Effect

The effects of different cations of ionic liquid aqueous solutions on extraction yield of shikimic acid were shown in Figure 2(b). With different cations from [C_2_mim]^+^ to [C_10_mim]^+^, the extraction yield of shikimic acid increased rapidly and then decreased with the extension of the carbon chain length and reached its maximum at [C_5_mim]^+^. Shikimic acid is water-soluble compounds and its skeleton consists of C–H. A range of carbon chain length of ionic liquids can help its dissolution; however, the hydrophobicity of an ionic liquid increases with the length of the alkyl chains on the imidazolium ring increasing, as shown elsewhere [43, 44], which leads to reduced solubility of shikimic acid. In addition, although [Bzmim]Br only has a 7 carbon aromatic chain cation the results demonstrated that shikimic acid extraction yield obtained by this ionic liquid was significantly higher than those observed with [C_6_mim]^+^ and [C_8_mim]^+^ (6–8 carbon chain cation) and slightly higher than the extraction yield observed with [C_5_mim]^+^ (5 carbon chain cation). This may be because the unsaturated shikimic acid is better dissolved by an unsaturated ionic liquid. [Bzmim]Br ionic liquid may be able to aid expansion or dissolution of cellulose and facilitate dissolution of shikimic acid from plant cells. Taking both the anion and cation of the ionic liquid into account, [Bzmim]Br aqueous solution was selected as extraction solvent for shikimic acid fromconifer needles.

#### 3.1.3. Concentration Effect

The optimum [Bzmim]Br concentration in aqueous solution for ILUAE of shikimic acid extraction was sought by carrying out extractions with [Bzmim]Br solutions of different concentrations (from 0.00 mol/L to 2 mol/L). Based on the results shown in Figure 2(c), it can be seen that the yield is increased with the [Bzmim]Br concentration ranged from 0.00 to 0.5 mol/L. However, a slight decrease in extraction yield was observed with further increases of [Bzmim]Br concentration. We proposed that the high viscosity of the solvent at high ionic liquid concentrations may lead to poor penetration of the solvent into the plant tissue [45], resulting in decreased extraction yield. Thus, 0.5 mol/L was therefore selected as the acceptable concentration level.

### 3.2. Optimization of the UAE Parameters

#### 3.2.1. Single Factor Experiments

The univariate method was used to optimize the following parameters: ultrasound power, ultrasound time, and solid-liquid ratio. The influences of each factor were studied by single-factor experiments.

*Effect of Ultrasound Time.* Ultrasound time is an important factor that would influence the extraction yield. Extraction experiments were carried out at different time conditions while other extraction parameters were 0.5 mol/L [Bzmim]Br, liquid-solid ratio 10 : 1 (mL/g), and ultrasound power 250 W. The effects of different times on extraction yield of shikimic acid were shown in Figure 2(d), which clearly indicated that the extraction yield increased rapidly in the first 30 min of sonication and the raise was then leveled when sonication was prolonged. Ultrasound facilitated the release of shikimic acid inside the plant cells to the exterior solvent and gave a large yield at the early stage of extraction. The rising effect may release most of the shikimic acid from the broken cells at the first 30 min; meanwhile, no obvious extraction yield change was observed in the prolonged time periods. Based on these results, 20–40 min ultrasound time was selected in the following experiments.

*Effect of Ultrasound Power*. The ultrasound power is very important to ensure an efficient extraction and the effect of this variable was examined. The different ultrasound powers such as 100, 150, 200, and 250 W were controlled. The rest of the variables employed were 0.5 mol/L [Bzmim]Br, liquid-solid ratio 10 : 1 (mL/g), and ultrasound time 30 min. Ultrasound power is believed to be the driving force for the complete dispersion of ionic liquid into the solid sample. From Figure 2(e), it can be observed that the extraction yield of shikimic acid which was greatly influenced by the ultrasound power increased sharply with the increase of ultrasound power up to 200 W, which may be due to the fact that it needs higher ultrasound power to ensure the dispersion of ionic liquid into the solid sample, and increased slowly as the ultrasound power increased further. Therefore, the 200 W ultrasound power could satisfy the conditions of high efficiency and low energy consumption. A 150–250 W ultrasound power was selected for subsequent experiments.

*Effect of Liquid-Solid Ratio. *In general, a higher solvent volume can dissolve target compound more effectively and result in a better extraction yield. For investigating the influence of liquid-solid ratio on extraction yield of shikimic acid, several tests were performed at different liquid-solid ratios with other extraction parameters being 0.5 mol/L [Bzmim]Br, ultrasound time 30 min, and ultrasound power 250 W. In this work, the liquid-solid ratios were investigated in the range of 6 : 1–20 : 1 mL/g. As shown in Figure 2(f), the extraction yield was promoted when the liquid-solid ratio was increased up to 10 : 1 mL/g and then increased slowly as the ratio increased further. Large solvent volume could make the procedure difficult and lead to unnecessary waste, while small volume may lead to incomplete extraction. For commercial application, a liquid-solid ratio between 8 and 12 mL/g should be optimized to avoid waste of solvent and bulky handling in the subsequent processes.

#### 3.2.2. Parameter Optimization by Response Surface Methodology

To further study the interactions between the factors, some parameters including ultrasound time, ultrasound power, and liquid-solid ratio were optimized using Box-Behnken design (BBD). As shown in Table 1, a model* F* value of 4.63 indicated that the model was significant, and there was only a 2.78% chance that a model* F* value of this size could occur due to statistical noise. Values of “probability >* F*” less than 0.0500 and greater than 0.1000 demonstrated that the model terms were significant and not significant, respectively. In this case, *X*_1_, *X*_1_ *X*_2_, and *X*_2_ *X*_3_ were significant model terms. A “pack of fit* P* value” of 0.3186 implied that the “lack of fit” was not significant. From Table 1, the negative “predicted *R* ^2^” implied that the overall mean was a better predictor. In “adequacy precision,” a signal-to-noise ratio of 7.6139 indicated an adequate signal. This model could be used to navigate the design space. The yield of shikimic acid (*Y*) was given by the following equation:

(3) Y=20.876+0.267X1−0.012X2−1.578X3  −0.002X1X2−0.014X1X3+0.013X2X3  +0.005X12−0.028X32.

**Figure 3:**
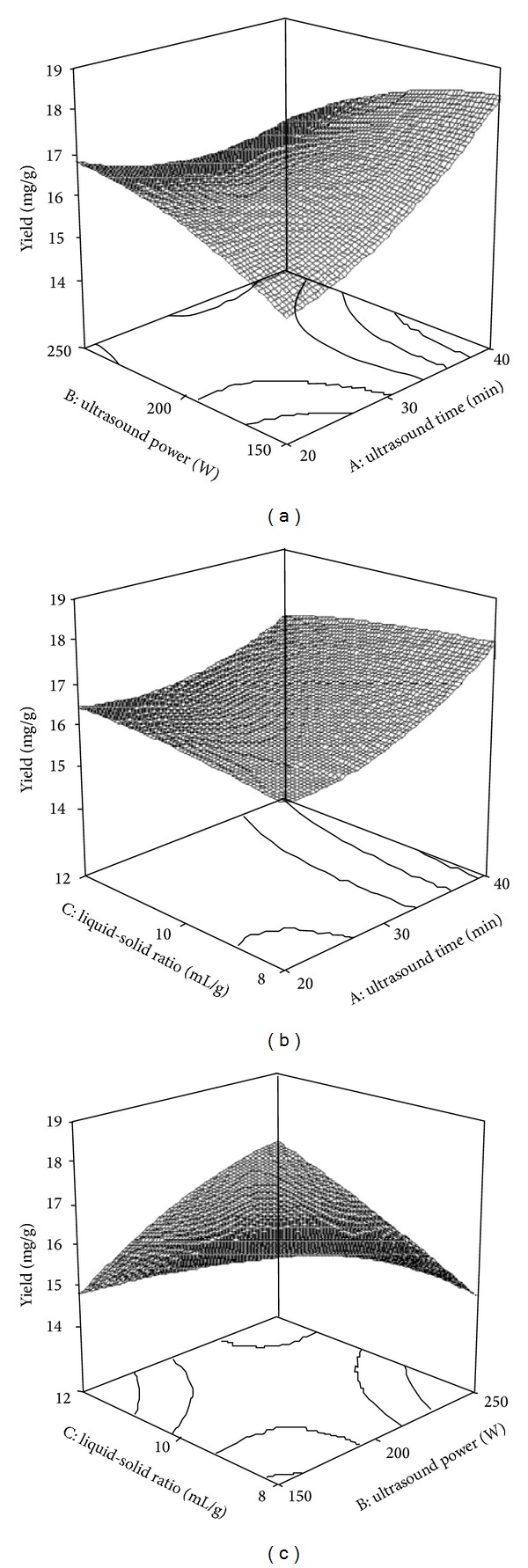
Response surface plots showing the effects of variables on extraction yield of shikimic acid. (a) Interaction of ultrasound time and ultrasound power; (b) interaction of ultrasound time and liquid-solid ratio; (c) interaction of ultrasound power and liquid-solid ratio.

Response surfaces were plotted to study the effects of parameters and their interactions on extraction yield. These three-dimensional response surface plots which show effects of two factors on the extraction yield with the third factor fixed were presented in Figure 3.  Figure 3(a) was the response surface and contour plot showing the effect of ultrasound time (*X*_1_) and ultrasound power (*X*_2_) on the yield at the fixed liquid-solid ratio. Both ultrasound time and power had positive effects on the extraction yield. It can be seen that the shikimic acid yield increased with the ultrasound power increase and then reached a maximum value, but the further increase of ultrasound power had a slightly effect on the yield. The yield increased rapidly with the increase of ultrasound time and reached a maximum value in the setpoint range.  Figure 3(b) depicted the interaction effect of ultrasound time (*X*_1_) and liquid-solid ratio (*X*_3_) on the yield at the fixed value of ultrasound power. The increase of ultrasound time could significantly enhance the yield, and then a maximum yield was obtained. However, a slightly effect of liquid-solid ratio on yield was observed.  Figure 3(c) described the interaction effect of ultrasound power (*X*_2_) and liquid-solid ratio (*X*_3_) on the yield when the ultrasound time was fixed. The optimum conditions for point prediction by software were as follows: 39 min ultrasound time, 170 W ultrasound power, and 8.3 : 1 liquid-solid ratio (mL/g). Under the conditions of point prediction, the extraction yield reached 18.78 mg/g.

#### 3.2.3. Verification Tests

The verification tests were done three times under the optimum conditions (0.5 mol/L [Bzmim]Br, 170 W ultrasound power, 39 min ultrasound time, and liquid-solid ratio of 8.3 : 1 mL/g). The actual extraction yield was 18.85 ± 0.78 mg/g.

### 3.3. Method Validation

To evaluate the proposed ILUAE approach, some parameters such as linearity, LOD, LOQ, reproducibility, stability, and recovery were determined under the above optimized conditions.

#### 3.3.1. Linearity, Limit of Detection, and Quantification

Calibration curves were obtained by plotting the peak area of the shikimic acid (*Y*) versus concentration (*X*) of shikimic acid. The regression linear equation of the calibration curve for shikimic acid was *Y* = (31577679 ± 309082)*X* + (613614 ± 7695) (*R* ^2^ = 0.9997,* n* = 6). The calibration curve showed good linearity for shikimic acid between 0.010 and 1.000 mg/mL. The LOD and LOQ for the determination of shikimic acid were 0.8 *μ*g/mL and 2.4 *μ*g/mL, respectively.

#### 3.3.2. Stability

The stability of shikimic acid in [Bzmim]Br solution was evaluated by determining standard solution of shikimic acid after ILUAE and one week later. The recovery of shikimic acid was taken to evaluate the stability of shikimic acid at the obtained operating extraction conditions. As shown in Table 2, the results indicated that complete recovery at the operating extraction conditions was 100.48% for standard solution of shikimic acid with no change in retention time of shikimic acid. After one week, the recovery of shikimic acid was 99.05%, which indicated that shikimic acid was stable in [Bzmim]Br solution.

#### 3.3.3. Recovery

To evaluate the accuracy of the proposed method, standard solution of shikimic acid was added to* P. meyeri* samples, at three levels, respectively. Under optimum conditions, samples with added standard solution were extracted by ILUAE, respectively, and then determined by HPLC detection to examine the recovery of the promoted method. The satisfactory results which were shown in Table 2 indicated that the mean recovery of shikimic acid from* P. meyeri* samples was 99.73%.

#### 3.3.4. Repeatability

To assess the repeatability of the promoted method, five extraction solutions of the* P. meyeri* samples were made by optimum ILUAE method. The average extraction yield of shikimic acid showed good repeatability with 0.93% of RSD. The results suggested that shikimic acid was stable in the ionic liquid solution and in the extracts. These method validation studies indicated that the proposed method is credible.

### 3.4. Comparison with the Reference and Conventional Methods

The reference methods tested included pure water extraction, 80% ethanol extraction, and sodium bromide solution extraction. Water is the most common and inexpensive solvent and is therefore often selected as a cosolvent in various extraction process. As can be seen from Table 3, the yield of the shikimic acid was only 15.41 ± 0.56 mg/g with pure water, while that obtained when using 0.5 mol/L [Bzmim]Br was 18.85 ± 0.78 mg/g. Therefore, we can conclude that the main contributor to shikimic acid extraction yield was the ionic liquid rather than water in the ionic liquid-water system through comparing the extraction capacities of [Bzmim]Br solution with pure water. The solvent effect of the ionic liquid was therefore more important in achieving high extraction yield than the salt effect derived from NaBr because the shikimic acid extraction yield achieved using 0.5 mol/L NaBr solution was only 14.56 ± 0.72 mg/g. Hence, salt effects could not play a major role in improving the extraction yield of shikimic acid. The shikimic acid extraction yield achieved using 80% ethanol solution was 17.36 ± 0.65 mg/g, lower than that obtained by 0.5 mol/L [Bzmim]Br solution. Therefore, 0.5 mol/L [Bzmim]Br solution was the best extraction solvent through comparing the yield of shikimic acid with other extraction solvents.

UAE and HRE methods were compared in the extraction of shikimic acid from* P. meyeri* in the current study. The yields of the shikimic acid obtained under optimal conditions of different extraction methods were summarized in Table 3. The conditions for UAE were that the extraction time was 39 min and extraction temperature was normal temperature. For HRE, the extraction time was 180 min and the extraction temperature was 85°C for 80% ethanol and 100°C for pure water, respectively. The shikimic acid extraction yield achieved using ILUAE method was higher than those obtained using other methods (Table 3). Hence, compared with regular HRE and UAE methods, the proposed ILUAE approach which used only small amount of ionic liquids could obtain a higher extraction yield in a shorter extraction time, which indicated that the [Bzmim]Br was an excellent extractant and ILUAE was an effective and rapid method for preparation of shikimic acid from conifer needles.

### 3.5. Actual Samples Determination

In order to obtain new plant resources of high shikimic acid content, shikimic acid contents in 85 conifer needles from different regions in China were investigated. The optimum ILUAE extraction condition obtained above was adopted and the result of shikimic acid contents was summarized in Table S1 in the Supporting Information.

#### 3.5.1. Shikimic Acid Contents in Conifer Needles from Different Regions in China

**Figure 4:**
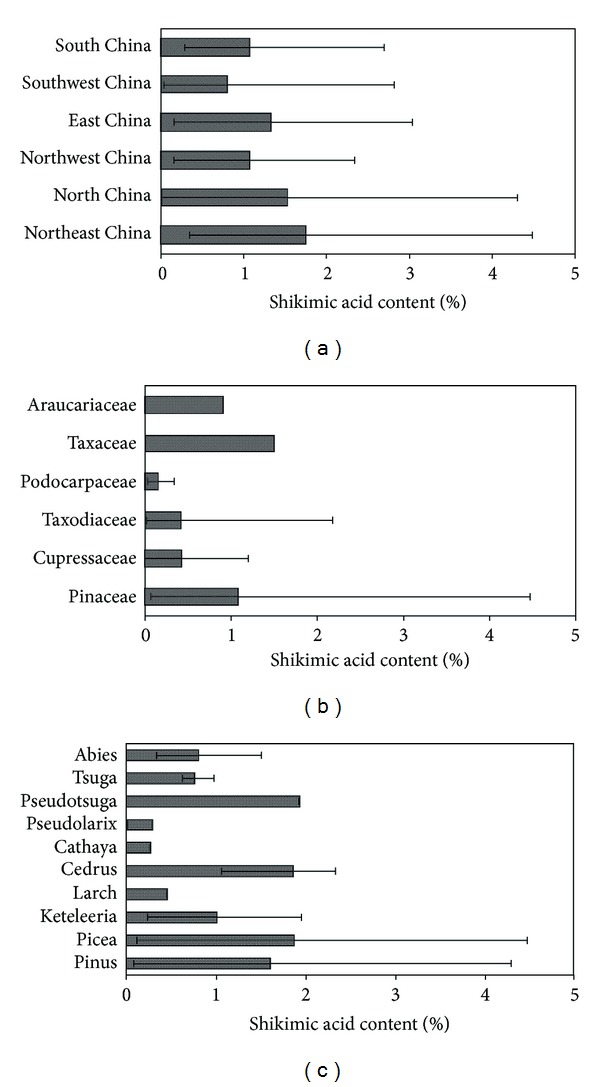
Shikimic acid content in conifer needles from (a) different regions in China; (b) different families; (c) different genera of Pinaceae.

By the measurement of shikimic acid, we got different contents of shikimic acid in 85 conifer needles, which were collected from the different regions of China in autumn 2011. As shown in Figure 4(a), the shikimic acid contents of different regions had great differences; even for each region, the shikimic acid contents of samples had distinct variances. For example, the Northeast had the highest shikimic acid content and also had the biggest standard deviation, the maximum value of shikimic acid content reached 4.47%, which was 13.55 times of the minimum value; the same situation also happened in the other regions. The highest content of shikimic acid was in the Northeast and followed by the North, and the lowest content of shikimic acid was in the South and the Northwest. Thus the Northeast and the North regions with lower annual average temperature were suitable for the production of shikimic acid in conifer needles which may be due to the cool climate.

#### 3.5.2. Shikimic Acid Contents in Conifer Needles from Different Families

From Table S1 and Figure 4(b), shikimic acid contents of 85 collected conifer needles which belonged to 6 families (including Pinaceae, Cupressaceae, Taxodiaceae, Podocarpaceae, Taxaceae, and Araucariaceae) were compared. The sample numbers of Pinaceae, Cupressaceae, abd Taxodiaceae were more than others, which were 58, 12, and 9, respectively. From Figure 4(b), obvious differences of shikimic acid contents from different families can be observed and the conifer needles which contained the highest shikimic acid content belonged to Pinaceae. It proposed that conifer needles of Pinaceae were better resources for extraction of shikimic acid.

#### 3.5.3. Shikimic Acid Contents in Conifer Needles of Different Genera from Pinaceae

The 58 kinds of conifer needles of Pinaceae were classified into 10 genera, including Pinus, Picea, Keteleeria, Larch, Cedrus, Cathaya, Pseudolarix, Pseudotsuga, Tsuga, and Abies, for the comparison of content differences of shikimic acid and the sample numbers of each genus were 27, 10, 6, 1, 3, 1, 1, 1, 3, and 5, respectively. Based on the results of Figure 4(c), a number of plant species, namely,* Picea koraiensis*,* Picea meyeri*,* Pinus elliottii,* and* Pinus banksiana*, were identified as among the best sources of shikimic acid.

Based on the above results, we found some distribution rules of shikimic acid in the conifer needles, but, till now, less physiological mechanisms were found about that. The further research will focus on the route of shikimic acid accumulation, and then fine academic explanation will be given to the distribution rules.

## 4. Conclusions

In this work, ultrasound-assisted extraction of shikimic acid using ionic liquid solution from conifer needles was investigated. As a kind of relative green solvent, ionic liquids were successfully used in the ILUAE procedure for the extraction of shikimic acid. With the addition of ionic liquids, the extraction yield of shikimic acid was improved greatly. The structure of ionic liquids, especially the cations, has a significant impact on the extraction yield of the target ingredient. The optimum ILUAE conditions were extracted with 0.5 mol/L [Bzmim]Br, liquid-solid ratio of 8.3 : 1 (mL/g), and 39 min ultrasound time under the power of 170 W. No degradation of the target analyte was observed under the optimum conditions, as evidenced from the stability studies performed with standard shikimic acid. The proposed method also shows high reproducibility. Under this condition, satisfactory extraction yield of the shikimic acid was obtained. Relative to other methods, the proposed approach provided higher extraction yield and obviously reduced energy consumption time. The present study provided useful information for acquisition and application of the shikimic acid resources.

## Supplementary Material

The shikimic acid contents of 85 kinds of conifer needles collected from six regions of China, including Northeast, North, South, Northwest, Southwest, East, were investigated. These conifer needles belong to various families (including Pinaceae, Cupressaceae, Taxodiaceae, Podocarpaceae, Taxaceae and Araucariaceae) and different genera of each family. Large amounts of information for selection a suitable conifer needle with high shikimic acid content in different places were listed in Table S1.

## Figures and Tables

**Table 1:** Experimental design matrix to screen for variables that determine the extraction yield of shikimic acid from *Picea meyeri* needles and ANOVA results^a^.

Run	BBD experiments				ANOVA					
	*X*_1_ (min)	*X*_2_ (W)	*X*_3_ (mL/g)	*Y* (mg/g)	Source	Sum of squares	Degree of freedom	Mean square	*F* value	*P* value
1	40	200	12	17.92	Model^b^	15.50	9	1.72	4.63	0.0278^c^
2	20	200	12	16.51	*X*_1_	3.80	1	3.80	10.21	0.0152^c^
3	30	200	10	16.80	*X*_2_	0.03	1	0.03	0.09	0.7761
4	20	150	10	15.37	*X*_3_	0.02	1	0.02	0.06	0.8184
5	40	250	10	16.20	*X*_1_ *X*_2_	3.37	1	3.37	9.06	0.0197^c^
6	40	200	8	17.97	*X*_1_ *X*_3_	0.30	1	0.30	0.80	0.4007
7	30	250	12	16.85	*X*_2_ *X*_3_	6.33	1	6.33	17.02	0.0044^c^
8	20	250	10	17.24	*X*_1_^2^	1.09	1	1.09	2.94	0.1302
9	20	200	8	15.47	*X*_2_^2^	0.59	1	0.59	1.59	0.2472
10	30	200	10	15.62	*X*_3_^2^	0.05	1	0.05	0.14	0.7200
11	30	150	8	17.83	Residual	2.60	7	0.37		
12	40	150	10	18.00	Lack of fit	1.43	3	0.48	1.62	0.3186
13	30	200	10	16.63	Pure error	1.18	4	0.29		
14	30	200	10	16.83	Cor. total	18.11	16			
15	30	150	12	14.62				
16	30	250	8	15.03						
17	30	200	10	16.97						

					Credibility analysis of the regression equations					
					Index mark	Standard deviation	Mean	Coefficient of variation%	*R* ^2^	Adequacy precision

					*Y*	0.61	16.58	3.68	0.8562	7.6139

^a^The results were obtained with Design Expert 7.0 software.

^b^ *X*_1_ is the ultrasound irradiation time (min), *X*_2_ is the ultrasound irradiation power (W), *X*_3_ is the liquid-solid ratio (mL/g), and *Y* is the extraction yield (mg/g).

^c^Significant at *P* < 0.05.

**Table 2:** Method validation studies.

Stability study of shikimic acid standard under the following ILUAE conditions: 170 W ultrasound power, 39 min ultrasound time, 8.3 : 1 liquid-solid ratio (mL/g), and 0.5 mol/L [Bzmim]Br as the extraction solvent						
Initial concentration (mg/mL)	Recovered concentration after ILUAE (mg/mL)	RSD% (*n* = 3)	Average recovery (%)	Recovered concentration after 7 days (mg/mL)	RSD% (*n* = 3)	Average recovery (%)

2.10	2.11	1.04	100.48	2.08	1.06	99.05

Recovery of shikimic acid from dried needle samples of *Picea meyeri* (*n* = 3)						

Sample	Shikimic acid content of the sample (mg)	Mass of added shikimic acid standard (mg)	Mass of the sample analyzed with added shikimic acid standard (mg)		Recovery (%)	

1	18.85	9.00	27.88		100.11	
2	18.85	18.03	36.77		99.70	
3	18.85	27.05	45.61		99.37	

Average					99.73	

**Table 3:** Comparison of ILUAE with other extraction methods, mean ± SD (*n* = 3).

Number	Solvent	Extraction method	Extraction time (min)	Extraction yield ± SD (mg/g)
1	Pure water	UAE	39	15.41 ± 0.56
2	Pure water	HRE	180	15.46 ± 0.49
3	80% ethanol	UAE	39	17.36 ± 0.65
4	80% ethanol	HRE	180	16.55 ± 0.75
5	0.5 mol/L NaBr	UAE	39	14.56 ± 0.72
6	0.5 mol/L [Bzmim]Br	UAE	39	18.85 ± 0.78

## References

[1] Widmer N, Meylan P, Ivanyuk A, Aouri M, Decosterd LA, Buclin T (2010). Oseltamivir in seasonal, avian H5N1 and pandemic 2009 AH1N1 influenza: pharmacokinetic and pharmacodynamic characteristics. *Clinical Pharmacokinetics*.

[2] Jiang M, Xiong B, Shen YM, Yang CH (2013). Design, synthesis, and preliminary biological evaluation of novel ketone derivatives of shikimic acid. *RSC Advances*.

[3] Tang L, Xiang H, Sun Y (2009). Monopalmityloxy shikimic acid: enzymatic synthesis and anticoagulation activity evaluation. *Applied Biochemistry and Biotechnology*.

[4] Xing J-F, Sun J-N, Sun J-Y (2012). Anti-inflammatory effect of 3,4-oxo-isopropylidene-shikimic acid on acetic acid-induced colitis in rats. *Digestive Diseases and Sciences*.

[5] Lin J, Lan Q, Wei YF, Liao LY, Wei GF (2008). An experimental study of the extraction procedure of medicinal components from star anise and its analgesic function. *Journal of Youjiang Medical College For Nationalities*.

[6] Chang Y-C, Almy EA, Blamer GA, Gray JI, Frost JW, Strasburg GM (2003). Antioxidant activity of 3-dehydroshikimic acid in liposomes, emulsions, and bulk oil. *Journal of Agricultural and Food Chemistry*.

[7] Aghil O, Bibby MC, Carrington SJ (1992). Synthesis and cytotoxicity of shikimate analogues. Structure: activity studies based on 1-crotonyloxymethyl-3R,4R,5R-trihydroxycyclohex-2-enone. *Anti-Cancer Drug Design*.

[8] Davies GM, Barrett-Bee KJ, Jude DA (1994). (6S)-6-fluoroshikimic acid, an antibacterial agent acting on the aromatic biosynthetic pathway. *Antimicrobial Agents and Chemotherapy*.

[9] Thayer A (2006). New routes to Tamiflu emerge. *Chemical & Engineering News*.

[10] Eykman JF (1885). Sur les principes constituants de *l'Illicium religiosum* (Sieb.) (Shikimi-no-ki en japonais). *Recueil Des Travaux Chimiques Des Pays-Bas*.

[11] Payne R, Edmonds M (2005). Isolation of shikimic acid from star aniseed. *Journal of Chemical Education*.

[12] Estévez AM, Estévez RJ (2012). A short overview on the medicinal chemistry of (-)-shikimic acid. *Mini-Reviews in Medicinal Chemistry*.

[13] Zhou B, Xu J Production preparation process of high-purity shikimic acid.

[14] Yang L, Huang J, Liu T (2010). Extraction technology of shikimic acid homogenate from *Picea koraiensis* optimized by response surface methodology. *Forest Engineering*.

[15] Ohira H, Torii N, Aida TM, Watanabe M, Smith RL (2009). Rapid separation of shikimic acid from Chinese star anise (*Illicium verum* Hook. f.) with hot water extraction. *Separation and Purification Technology*.

[16] Harring T, Streibig JC, Husted S (1998). Accumulation of shikimic acid: a technique for screening glyphosate efficacy. *Journal of Agricultural and Food Chemistry*.

[17] Mueller TC, Massey JH, Hayes RM, Main CL, Stewart CN (2003). Shikimate accumulates in both glyphosate-sensitive and glyphosate-resistant horseweed (*Conyza canadensis* L. Cronq.). *Journal of Agricultural and Food Chemistry*.

[18] Anderson KA, Cobb WT, Loper BR (2001). Analytical method for determination of shikimic acid: shikimic acid proportional to glyphosate application rates. *Communications in Soil Science and Plant Analysis*.

[19] Jaroszyńska J (2003). Isolation of free phenolic compounds from arboreal leaves by use of the Florisil/C_18_ system. *Analytical and Bioanalytical Chemistry*.

[20] Feng S-X, Guo H-Z, Liu P, Li H-D, Guo D-A (2008). Chemical constituents in bark of *Pseudolarix kaempferi*. *Chinese Traditional and Herbal Drugs*.

[21] Iyer SV, Pejakala V, Karabasanagouda Method for obtaining shikimic acid.

[22] Deng Y Preparation of shikimic acid.

[23] Ma C-H, Liu T-T, Yang L, Zu Y-G, Wang S-Y, Zhang R-R (2011). Study on ionic liquid-based ultrasonic-assisted extraction of biphenyl cyclooctene lignans from the fruit of Schisandra chinensis Baill. *Analytica Chimica Acta*.

[24] Yang L, Wang H, Zu Y-G (2011). Ultrasound-assisted extraction of the three terpenoid indole alkaloids vindoline, catharanthine and vinblastine from *Catharanthus roseus* using ionic liquid aqueous solutions. *Chemical Engineering Journal*.

[25] Yang L, Liu Y, Zu Y-G (2011). Optimize the process of ionic liquid-based ultrasonic-assisted extraction of aesculin and aesculetin from *Cortex fraxini* by response surface methodology. *Chemical Engineering Journal*.

[26] Yang L, Li L, Liu T (2013). Development of sample preparation method for isoliquiritigenin, liquiritin, and glycyrrhizic acid analysis in licorice by ionic liquids- ultrasound based extraction and high-performance liquid chromatography detection. *Food Chemistry*.

[27] Yang L, Ge H, Wang W (2013). Development of sample preparation method for eleutheroside B and E analysis in *Acanthopanax senticosus* by ionic liquids- ultrasound based extraction and high-performance liquid chromatography detection. *Food Chemistry*.

[28] Zu G, Zhang R, Yang L (2012). Ultrasound assisted extraction of carnosic acid and rosmarinic acid using ionic liquid solution from *Rosmarinus officinalis*. *International Journal of Molecular Sciences*.

[29] Sun X, Jin Z, Yang L (2013). Ultrasonic-Assisted extraction of procyanidins using ionic liquid solution from *Larix gmelinii* Bark. *Journal of Chemistry*.

[30] Berthod A, Ruiz-Ángel MJ, Carda-Broch S (2008). Ionic liquids in separation techniques. *Journal of Chromatography A*.

[31] Ruiz-Aceituno L, Sanz ML, Ramos L (2013). Use of ionic liquids in analytical sample preparation of organic compounds from food and environmental samples. *TrAC Trends in Analytical Chemistry*.

[32] Liu T, Sui X, Zhang R (2011). Application of ionic liquids based microwave-assisted simultaneous extraction of carnosic acid, rosmarinic acid and essential oil from *Rosmarinus officinalis*. *Journal of Chromatography A*.

[33] Trujillo-Rodríguez MJ, Rocío-Bautista P, Pino V, Afonso AM (2013). Ionic liquids in dispersive liquid-liquid microextraction. *TrAC Trends in Analytical Chemistry*.

[34] Vidal L, Riekkola M-L, Canals A (2012). Ionic liquid-modified materials for solid-phase extraction and separation: a review. *Analytica Chimica Acta*.

[35] Wang S-Y, Yang L, Zu Y-G (2011). Design and performance evaluation of ionic-liquids-based microwave-assisted environmentally friendly extraction technique for camptothecin and 10-hydroxycamptothecin from samara of camptotheca acuminata. *Industrial and Engineering Chemistry Research*.

[36] Ma C-H, Wang S-Y, Yang L (2012). Ionic liquid-aqueous solution ultrasonic-assisted extraction of camptothecin and 10-hydroxycamptothecin from Camptotheca acuminata samara. *Chemical Engineering and Processing*.

[37] Yang L, Sun X, Yang F, Zhao C, Zhang L, Zu Y (2012). Application of ionic liquids in the microwave-assisted extraction of proanthocyanidins from *larix gmelini* bark. *International Journal of Molecular Sciences*.

[38] Liu Y, Yang L, Zu Y (2012). Development of an ionic liquid-based microwave-assisted method for simultaneous extraction and distillation for determination of proanthocyanidins and essential oil in *Cortex cinnamomi*. *Food Chemistry*.

[39] Ma C-H, Liu T-T, Yang L (2011). Ionic liquid-based microwave-assisted extraction of essential oil and biphenyl cyclooctene lignans from *Schisandra chinensis* Baill fruits. *Journal of Chromatography A*.

[40] Usuki T, Yasuda N, Yoshizawa-Fujita M, Rikukawa M (2011). Extraction and isolation of shikimic acid from *Ginkgo biloba* leaves utilizing an ionic liquid that dissolves cellulose. *Chemical Communications*.

[41] Anderson JL, Ding J, Welton T, Armstrong DW (2002). Characterizing ionic liquids on the basis of multiple solvation interactions. *Journal of the American Chemical Society*.

[42] Huddleston JG, Visser AE, Reichert WM, Willauer HD, Broker GA, Rogers RD (2001). Characterization and comparison of hydrophilic and hydrophobic room temperature ionic liquids incorporating the imidazolium cation. *Green Chemistry*.

[43] Lu Y, Ma W, Hu R, Dai X, Pan Y (2008). Ionic liquid-based microwave-assisted extraction of phenolic alkaloids from the medicinal plant *Nelumbo nucifera* Gaertn. *Journal of Chromatography A*.

[44] Bonhote P, Dias A-P, Papageorgiou N, Kalyanasundaram K, Graetzel M (1996). Hydrophobic, highly conductive ambient-temperature molten salts. *Inorganic Chemistry*.

[45] Du F-Y, Xiao X-H, Li G-K (2007). Microwave-assisted extraction of alkaloids in *Lycoris Radiata* using ionic liquids solution. *Chinese Journal of Analytical Chemistry*.

