# Supplementary material for: Extraction and Chromatographic Determination of Shikimic Acid in Chinese Conifer Needles with 1-Benzyl-3-methylimidazolium Bromide Ionic Liquid Aqueous Solutions
Source: J Anal Methods Chem. 2014 Mar 23;2014:256473. doi: 10.1155/2014/256473 (PMC3980885; doi:10.1155/2014/256473)
Supplement: Supplementary file 1 — The shikimic acid contents of 85 kinds of conifer needles collected from six regions of China, including Northeast, North, South, Northwest, Southwest, East, were investigated. These conifer needles belong to various families (including Pinaceae, Cupressaceae, Taxodiaceae, Podocarpaceae, Taxaceae and Araucariaceae) and different genera of each family. Large amounts of information for selection a suitable conifer needle with high shikimic acid content in different places were listed in Table S1. [file 256473.f1.doc]

**Table S1** shikimic acid content of 85 kinds of conifer needles in different regions of China (n=6)

| No. | Sampling position (annual average temperature, ºC; Annual average precipitation, mm) | Species name | Family | Content% | +SD | -SD |
| --- | --- | --- | --- | --- | --- | --- |
| Beijing Botanical Garden (Beijing, North China), (12; 626) | | | | | | |
| 1 |  | *Thnja koraicnsis* | Cupressaceae | 0.00 | 0.00 | 0.00 |
| 2 |  | *Pinus koraiensis* | Pinaceae | 1.59 | 0.05 | 0.09 |
| 3 |  | *Juniperus chinensis* | Cupressaceae | 0.00 | 0.00 | 0.00 |
| 4 |  | *Picea koraiensis* *var intercedens* | Pinaceae | 1.97 | 0.07 | 0.08 |
| 5 |  | *Pinus pumila* | Pinaceae | 0.32 | 0.09 | 0.04 |
| 6 |  | *Pinus takahasii* | Pinaceae | 2.52 | 0.14 | 0.08 |
| 7 |  | *Picea wilsonii* | Pinaceae | 2.32 | 0.09 | 0.19 |
| 8 |  | *Abies nephrolepis* | Pinaceae | 1.10 | 0.02 | 0.07 |
| 9 |  | *Pinus thunbergii* | Pinaceae | 2.26 | 0.28 | 0.04 |
| 10 |  | *Pinus sylvesris var mongolica* | Pinaceae | 1.60 | 0.20 | 0.51 |
| 11 |  | *Picea koraiensis* | Pinaceae | 2.46 | 0.01 | 0.11 |
| 12 |  | *Pinus banksiana* | Pinaceae | 4.29 | 0.09 | 0.01 |
| 13 |  | *Sabina chinensis* | Cupressaceae | 0.00 | 0.16 | 0.16 |
| 14 |  | *Abies holophylla* | Pinaceae | 0.64 | 0.15 | 0.13 |
| Qinling National Botanical Garden (Qinling,Northwest China), (14; 850) | | | | | | |
| 15 |  | *Pinus bungeana* | Pinaceae | 1.61 | 0.15 | 0.31 |
| 16 |  | *Picea asperata* | Pinaceae | 0.14 | 0.27 | 0.07 |
| 17 |  | *Picea wilsonii* | Pinaceae | 1.79 | 0.28 | 0.44 |
| 18 |  | *Cryptomeria fortunei* | Taxodiaceae | 0.31 | 0.40 | 0.07 |
| 19 |  | *Pinus tabulaeformis* | Pinaceae | 0.69 | 0.12 | 0.44 |
| 20 |  | *Cedrus deodara* | Pinaceae | 2.33 | 0.38 | 0.08 |
| 21 |  | *Platycladus orientalis* | Cupressaceae | 0.77 | 0.19 | 0.34 |
| 22 |  | *Juniperus formosana* | Cupressaceae | 0.97 | 0.08 | 0.24 |
| 23 |  | *Juniperus chinensis cv. kaizuka* | Cupressaceae | 0.69 | 0.08 | 0.09 |
| 24 |  | *Juniperus chinensis* | Cupressaceae | 0.52 | 0.15 | 0.05 |
| 25 |  | *Sabina chinensis cv pyramidali* | Cupressaceae | 1.20 | 0.19 | 0.09 |
| 26 |  | *Taxus chinenwsis varmairei* | Taxaceae | 1.49 | 0.11 | 0.24 |
| Shanghai Botanical Garden (East China)(18; 1178) | | | | | | |
| 27 |  | *Podocaarpus macrophyllus* | Podocarpaceae | 0.15 | 0.01 | 0.22 |
| 28 |  | *Pinus elliottii* | Pinaceae | 3.02 | 0.08 | 0.01 |
| 29 |  | *Pinus parviflora* | Pinaceae | 2.07 | 0.02 | 0.08 |
| 30 |  | *Cedrus deodara* | Pinaceae | 1.05 | 0.29 | 0.02 |
| 31 |  | *Pinus thunbergii* | Pinaceae | 1.37 | 0.11 | 0.48 |
| 32 |  | *Cryptomeria japonica* | Taxodiaceae | 2.18 | 0.00 | 0.11 |
| 33 |  | *Juniperus chinensis cv kaizuka* | Cupressaceae | 0.22 | 0.03 | 0.01 |
| 34 |  | *Platycladus orientalis* | Cupressaceae | 0.32 | 0.01 | 0.02 |
| South China Botanical Garden (South China)(23; 1705) | | | | | | |
| 35 |  | *Podocaarpus macrophyllus* | Podocarpaceae | 0.34 | 0.01 | 0.14 |
| 36 |  | *Araucaria cunninghamia* | Araucariaceae | 0.89 | 0.02 | 0.01 |
| 37 |  | *Platycladus orientalis* | Cupressaceae | 0.27 | 0.29 | 0.02 |
| 38 |  | *Pinus massoniana* | Pinaceae | 2.68 | 0.11 | 0.11 |
| Tibet, Nyingchi Prefecture, in southern suburbs of Nyingchi city (Southwest China)(8.7; 650) | | | | | | |
| 39 |  | *Pinus armandii* | Pinaceae | 0.07 | 0.03 | 0.02 |
| 40 |  | *Picea likiangensis var. linzhiensis* | Pinaceae | 0.11 | 0.01 | 0.01 |
| 41 |  | *Pinus griffithii* | Pinaceae | 0.09 | 0.15 | 0.14 |
| Chuxiong Yi Autonomous Prefecture (Southwest China)(15.7; 850) | | | | | | |
| 42 |  | *Pinus armandii* | Pinaceae | 1.91 | 0.01 | 0.01 |
| 43 |  | *Keteleeria evelyniana* | Pinaceae | 1.95 | 0.15 | 0.02 |
| Kunming Botanical Garden (Southwest China)(20; 1101.7) | | | | | | |
| 44 |  | *Cathaya argyrophylla* | Pinaceae | 0.25 | 0.06 | 0.06 |
| 45 |  | *Keteleeria evelyniana* | Pinaceae | 0.75 | 0.11 | 0.09 |
| 46 |  | *Keteleeria fortunei* | Pinaceae | 1.32 | 0.05 | 0.05 |
| 47 |  | *Keteleeria pubesens* | Pinaceae | 0.86 | 0.12 | 0.08 |
| 48 |  | *Keteleeria cyclolepis* | Pinaceae | 0.23 | 0.07 | 0.04 |
| 49 |  | *Keteleeria calcarea* | Pinaceae | 0.84 | 0.09 | 0.08 |
| 50 |  | *Pinus fenzeliana* | Pinaceae | 1.29 | 0.14 | 0.19 |
| 51 |  | *Pinus armandii* | Pinaceae | 1.17 | 0.09 | 0.07 |
| 52 |  | *Pinus taeda* | Pinaceae | 1.02 | 0.28 | 0.51 |
| 53 |  | *Pinus kesiya var langbianensis* | Pinaceae | 1.04 | 0.20 | 0.11 |
| 54 |  | *Pinus pinaster* | Pinaceae | 0.33 | 0.01 | 0.01 |
| 55 |  | *Pinus yunnanensis* | Pinaceae | 0.14 | 0.09 | 0.16 |
| 56 |  | *Pinus pinea* | Pinaceae | 0.82 | 0.16 | 0.13 |
| 57 |  | *Pinus radiata* | Pinaceae | 2.80 | 0.15 | 0.15 |
| 58 |  | *Pinus sylvesris var mongolica* | Pinaceae | 2.80 | 0.17 | 0.31 |
| 59 |  | *Pinus elliottii* | Pinaceae | 0.75 | 0.15 | 0.07 |
| 60 |  | *Picea likiangensis* | Pinaceae | 1.22 | 0.27 | 0.44 |
| 61 |  | *Picea smithiana* | Pinaceae | 0.23 | 0.28 | 0.07 |
| 62 |  | *Abies forrestii* | Pinaceae | 1.50 | 0.40 | 0.44 |
| 63 |  | *Abies ernestii var salouenensis* | Pinaceae | 0.36 | 0.12 | 0.08 |
| 64 |  | *Cedrus deodara* | Pinaceae | 2.16 | 0.38 | 0.34 |
| 65 |  | *Pseudolarix kaempferi* | Pinaceae | 0.27 | 0.19 | 0.24 |
| 66 |  | *Tsuga chinensis var robusta* | Pinaceae | 0.97 | 0.08 | 0.09 |
| 67 |  | *Tsuga dumosa* | Pinaceae | 0.62 | 0.08 | 0.05 |
| 68 |  | *Tsuga forrestii* | Pinaceae | 0.65 | 0.15 | 0.09 |
| 69 |  | *Pseudotsuga sinensis* | Pinaceae | 1.92 | 0.19 | 0.24 |
| 70 |  | *Sequoia sempervirens* | Taxodiaceae | 0.22 | 0.11 | 0.10 |
| 71 |  | *Metasequoia glyptostroboides* | Taxodiaceae | 1.03 | 0.35 | 0.22 |
| 72 |  | *Cunninghamia lanceolata* | Taxodiaceae | 0.02 | 0.01 | 0.01 |
| 73 |  | *Sabina vulgaris* | Cupressaceae | 0.17 | 0.08 | 0.08 |
| 74 |  | *Taxodium distichum* | Taxodiaceae | 0.04 | 0.02 | 0.02 |
| 75 |  | *Taxodium mucronatum* | Taxodiaceae | 0.50 | 0.29 | 0.48 |
| 76 |  | *Cryptomeria fortunei* | Taxodiaceae | 0.15 | 0.11 | 0.11 |
| 77 |  | *Taiwania cryptomerioides* | Taxodiaceae | 0.05 | 0.00 | 0.01 |
| 78 |  | *Podocarpus macrophylla var maki* | Podocarpaceae | 0.04 | 0.03 | 0.02 |
| 79 |  | *Podocarpus macrophyllus var angustitolius* | Podocarpaceae | 0.03 | 0.01 | 0.01 |
| 80 |  | *Pinus bungeana* | Pinaceae | 0.22 | 0.15 | 0.14 |
| Harbin Experimental Forest Farm of Northeast Forestry University (Northeast China)(6; 500) | | | | | | |
| 81 |  | *Picea meyeri* | Pinaceae | 4.47 | 0.08 | 0.22 |
| 82 |  | *Picea koraiensis* | Pinaceae | 2.93 | 0.15 | 0.01 |
| 83 |  | *Larix gmellinii* | Pinaceae | 0.44 | 0.19 | 0.08 |
| 84 |  | *Pinus sylvesris var mongolica* | Pinaceae | 0.44 | 0.11 | 0.02 |
| 85 |  | *Abies holophylla* | Pinaceae | 0.33 | 0.35 | 0.48 |
